# Memory Effect on the Survival of Deinococcus radiodurans after Exposure in Near Space

**DOI:** 10.1128/spectrum.03474-22

**Published:** 2023-02-07

**Authors:** Yining Chen, Qing Zhang, Deyu Wang, Yao-Gen Shu, Hualin Shi

**Affiliations:** a CAS Key Laboratory of Theoretical Physics, Institute of Theoretical Physics, Chinese Academy of Sciences, Beijing, China; b School of Physical Sciences, University of Chinese Academy of Sciences, Beijing, China; c Wenzhou Institute, University of Chinese Academy of Sciences, Wenzhou, China; University of Minnesota Twin Cities

**Keywords:** Earth’s near space, radiation resistance, astrobiology, *Deinococcus radiodurans*

## Abstract

Near space (20 to 100 km in altitude) is an extreme environment with high radiation and extreme cold, making it diﬃcult for organisms to survive. However, many studies had shown that there were still microbes living in this extremely harsh environment. It was particularly important to study which factors affected the survival of microorganisms living in near space after exposure to irradiation, as this was related to many studies, such as studies of radioresistance mechanisms, panspermia hypothesis, long-distance microbial transfer, and developing extraterrestrial habitats. Survival after radiation was probably influenced by the growth condition before radiation, which is called the memory effect. In this research, we used different growth conditions to affect the growth of Deinococcus radiodurans and lyophilized bacteria in exponential phase to maintain the physiological state at this stage. Then high-altitude scientific balloon exposure experiments were carried out by using the Chinese Academy of Sciences Balloon-Borne Astrobiology Platform (CAS-BAP) at Dachaidan, Qinghai, China (37°44′N, 95°21′E). The aim was to investigate which factors influence survival after near-space exposure. The results suggested that there was a memory effect on the survival of D. radiodurans after exposure. If the differences in growth rate were caused by differences in nutrition, the survival rate and growth rate were positively correlated. Moreover, the addition of paraquat and Mn^2+^ during the growth phase can also increase survival. This finding may help to deepen the understanding of the mechanics of radiation protection and provide relevant evidence for many studies, such as of long-distance transfer of microorganisms in near space.

**IMPORTANCE** Earth’s near space is an extreme environment with high radiation and extreme cold. Which factors affect the survival of microbes in near space is related to many studies, such as studies of radioresistance mechanisms, panspermia hypothesis, long-distance microbial transfer, and developing extraterrestrial habitats. We performed several exposure experiments with Deinococcus radiodurans in near space to investigate which factors influence the survival rate after near-space exposure; that is, there was a relationship between survival after radiation and the growth condition before radiation. The results suggested that there was a memory effect on the survival of D. radiodurans after exposure. This finding may help to deepen the understanding of the mechanism of radiation protection and provide relevant evidence for many studies, such as of long-distance transfer of microorganisms in near space.

## INTRODUCTION

Investigations of microbial behavior in extreme environments are drawing increasing attention, as it may reveal patterns of microbes that cannot be observed under current conditions and improve our understanding of extremophilic life. This will be of great significance for testing the panspermia hypothesis, assessing long-distance microbial transfer, and developing extraterrestrial habitats, as near spaces provides unique analogues for the Martian surface (1, 2). Near space (20 to 100 km in altitude) is such an extreme environment, with extreme cold and high radiation, which is the boundary of Earth’s biosphere (3). Previous literature has shown that several microbes have been found in near space. The usual explanation is that some microbes that live on Earth’s surface adhere to the dust and travel to near space with updrafts (4–7). These microbes endure extreme environments in near space, and some of them survive to drift to another location with air currents (3, 4, 8). Studying the survival of bacteria in near space helps to understand the mechanisms needed for surviving in high radiation, as well as to study the subsistence in harsh environments (9, 10). Experimentation in near space is low cost but eﬃcient because only a balloon is required to launch the device and samples, which is an inexpensive and short-cycle approach. The study of near-space microorganisms based on high-altitude balloons can be traced back to the 1930s (1, 6, 11, 12). Additionally, some of these resilient bacteria, such as Deinococcus radiodurans and Ramazzottius varieornatus, have been sent to the International Space Station (ISS), orbiting in the low Earth orbit (LEO), about 360 km in altitude, to study their behaviors in the LEO and show the probability of survival in the LEO (13–23). One of the main goals of these space experiments was to explore habitability and potential signs of life beyond Earth. The Balloon-Borne Astrobiology Platform in near space is such a scheme that not only provides a low-cost and effective alternative for astrobiological research but also could be combined with experiments in the LEO to form a complete space biological investigation (9, 24).

The harsh conditions for microorganisms in near space include very low atmospheric pressure, extremely low temperature, and high UV and cosmic radiation, of which low temperature and radiation are the main causes of death for microbes (6, 12, 19, 25). According to the survival experiments under simulated LEO conditions, UV radiation may be more fatal to aggregated bacteria than low temperature (3, 20, 26, 27). According to the previous literature, it was generally believed that the effectiveness of UV damage was caused by UVC light (200 to 280 nm) (28–30). Absorbed UVC radiation induces the oxidation of proteins and lipids, as well as various types of DNA damage, including the DNA photoproduct cyclobutan pyrimidine dimer (CPD) and DNA single-strand breaks (SSBs) and double-strand breaks (DSBs) (31, 32). During irradiation, the accumulation of DNA damages, oxidants, and damaged proteins cause an inhibition of transcription and replication, thereby preventing cell proliferation and leading to cell death (32). Bacteria can survive exposure of UV radiation by using repair systems which could be influenced by physiology depending on their growth conditions (28, 29, 32–35). Growth conditions can change the cellular composition and physiology, which directly affect the effectiveness of DNA repair mechanisms (36–44). Therefore, the relationship between growth conditions and survival of near-space exposure is worth studying (45).

A great opportunity to study this issue is provided by a certain prokaryote, which has been evolutionarily optimized for dealing with genotoxic agents and harsh environments such as UVC radiation, oxidative stress, and desiccation (46–48). The bacterium Deinococcus radiodurans, known for its extreme resistance to high doses of radiation, oxidative stress, and DNA damage as well as for its ability to repair massive DNA damage, including hundreds of DBSs, is the best-studied organism in this category. It can survive acute exposure to up to 5 kGy of gamma radiation and up to 1,000 J/m^2^ of UV radiation without loss of viability or mutation (47–54). The viability of D. radiodurans exposed to solar and cosmic radiations has been confirmed by the Exposure Facility of the Japanese Experimental Module (JEM) of the ISS during the space mission Tanpopo (18, 20, 21, 25, 55). This provides evidence that this bacterium can be used for experiments in near space.

The growth environment affects the physiology and gene expression of bacteria and may affect their survival in stressful environments; e.g., Escherichia coli with noisy growth modulation could survive stress better (56). When bacteria encounter a stressful environment, the survival rate depends not only on the type of bacteria but also on the environment they have experienced before. We call this phenomenon, which results in changes in survival rate in response to stress due to differences in physiology and gene expression caused by previous growth environments, the memory effect. With the aim of investigating the memory effect on microorganisms subjected to near-space exposure, we performed three exposure experiments in near space through the Biological Samples Exposure Payload (BIOSEP) carried by a high-altitude scientific balloon using the Chinese Academy of Sciences Balloon-Borne Astrobiology Platform (CAS-BAP) at Dachaidan, Qinghai, China (37°44′N, 95°21′E) from 2019 to 2021, named HH-19-9, HH-20-7, and HH-21-5 (24, 57). D. radiodurans bacteria growing in different environments were freeze-dried in the exponential phase and exposed to near space to determine the survival rate. We observed that there was a certain correlation between survival of exposure and growth environment. Then we performed experiments in the laboratory to verify our results. The details of experiments are described in Materials and Methods.

## RESULTS

### Effects of different growth rates on resistance of D. radiodurans to exposure.

**FIG 1:**
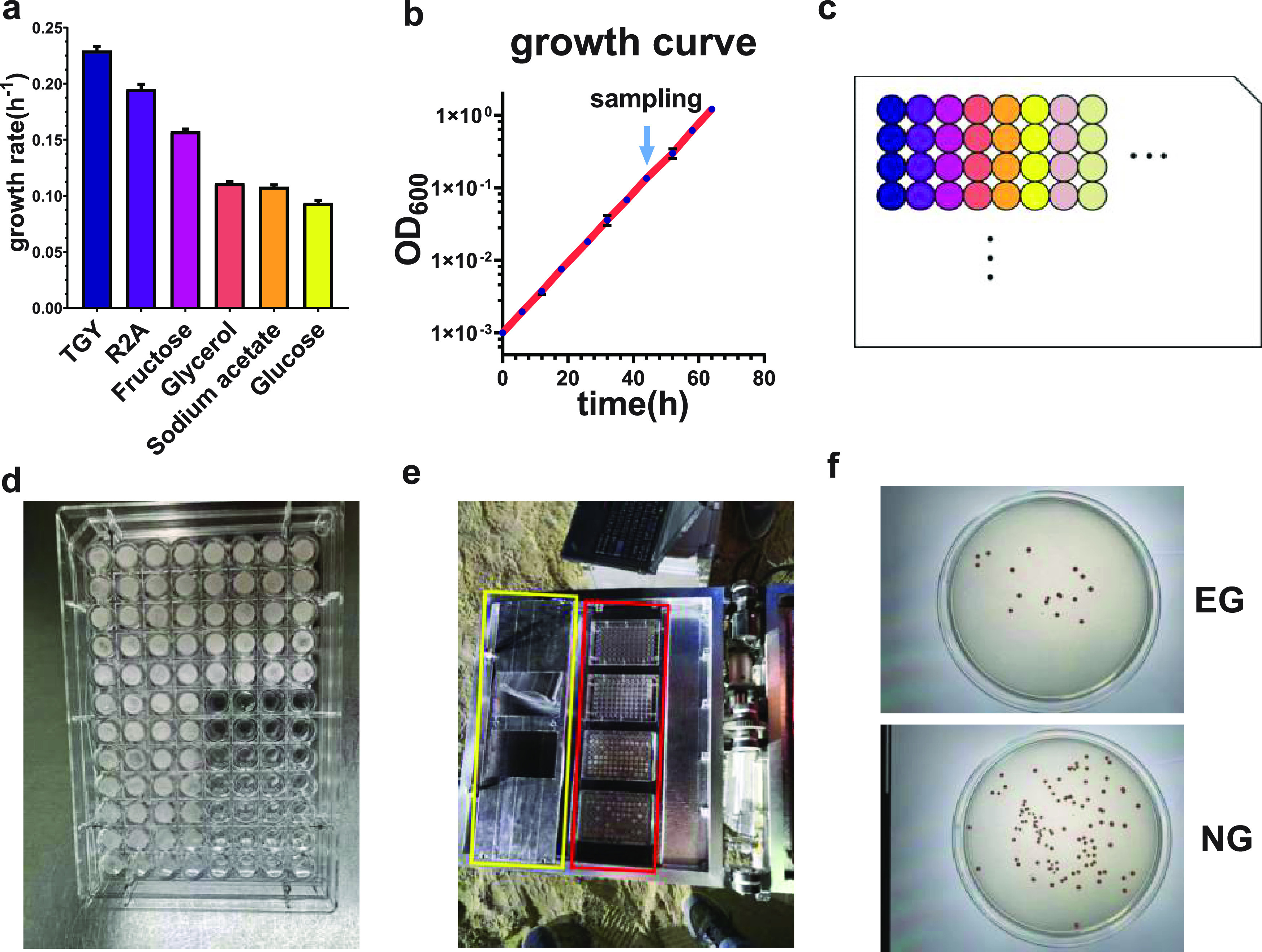
Preparation of exposure experiment in near space. (a) Growth rates of D. radiodurans in diverse media. (b) Growth curve of D. radiodurans growing with glycerol as carbon source. The light blue arrow shows the time point of sampling. (c) Sketch map of samples in 96-well plate. (d) Photo of one plate. The pink-orange substance in each well is a thin layer of lyophilized D. radiodurans. (e) Photo of BIOSEP with our samples fixed on it. The red box marks where we stored our samples, and the yellow box is the lid. The two square holes on the lid are for exposing the samples, and the part without holes is for shielding nonexposed samples. (f) Example of plates for counting CFU of D. radiodurans (dilution fold was 10^4^). EG, exposure group; NG, nonexposure group.

The effects of different growth rates on survival after exposure were investigated first. We used diverse media to control the growth rate of D. radiodurans (Fig. 1a and Table 1) (58). We cultivated bacteria to exponential phase and freeze-dried them in 96-well plates (Fig. 1b to d). The details of the experiments are shown in Materials and Methods. The bacteria in the exponential phase rather than the stationary phase were selected as samples because the former could better reflect the characteristics of different environments, while the most of bacteria in the latter were in a dormant state (36, 59). The samples were stored at −20°C before the experiment (Fig. 1e). Then the samples were fixed on BIOSEP and carried by a helium balloon which was launched into near space, stayed at the target altitude for a period of time for exposure, and landed at our planned location. (The exposure times were 2 h 22 min, 7 h 16 min, 2 h, and 4 h for HH-19-9, HH-20-7, HH-21-5-short, and HH-21-5-long, respectively.) After flight, the samples were stored at −20°C and brought back to the laboratory. The lyophilized samples were suspended into basal salt medium, and we determined the survival rates of bacteria by the colony-counting method (Fig. 1f) (24, 57).

**TABLE 1:** Growth media and conditions

Medium	Ingredient	Concn
TGY	Casein tryptone	0.3% (m/v)
	Yeast extract fermentation	0.25% (m/v)
	Glucose	0.1% (m/v)
** **		
R2A	Casein tryptone	0.05% (m/v)
	Yeast extract fermentation	0.05% (m/v)
	Casein hydrolysate	0.05% (m/v)
	Glucose	0.05% (m/v)
	Soluble starch	0.05% (m/v)
	Potassium dihydrogen phosphate	0.03% (m/v)
	Magnesium sulfate	0.0024% (m/v)
	Sodium pyruvate	0.03% (m/v)
** **		
Minimal nutrient medium	Potassium phosphate buffer (pH 7.5–8.0)	20 mM
	Magnesium chloride tetrahydrate	0.2 mM
	Calcium chloride dihydrate	0.1 mM
	Manganese(II) acetate tetrahydrate	5.0 μM
	Ammonium molybdate tetrahydrate	5.0 μM
	Ferrous sulfate heptahydrate	5.0 μM
	Nicotinic acid	1.0 μg/mL
	Casamino Acids	0.2% (m/v)
** **		
Extra reagent for pressure	Paraquat methosulfate (for oxidative stress)	10.0 μM
	Manganese(II) acetate tetrahydrate (for high level of Mn^2+^)	50.0 μM

**FIG 2:**
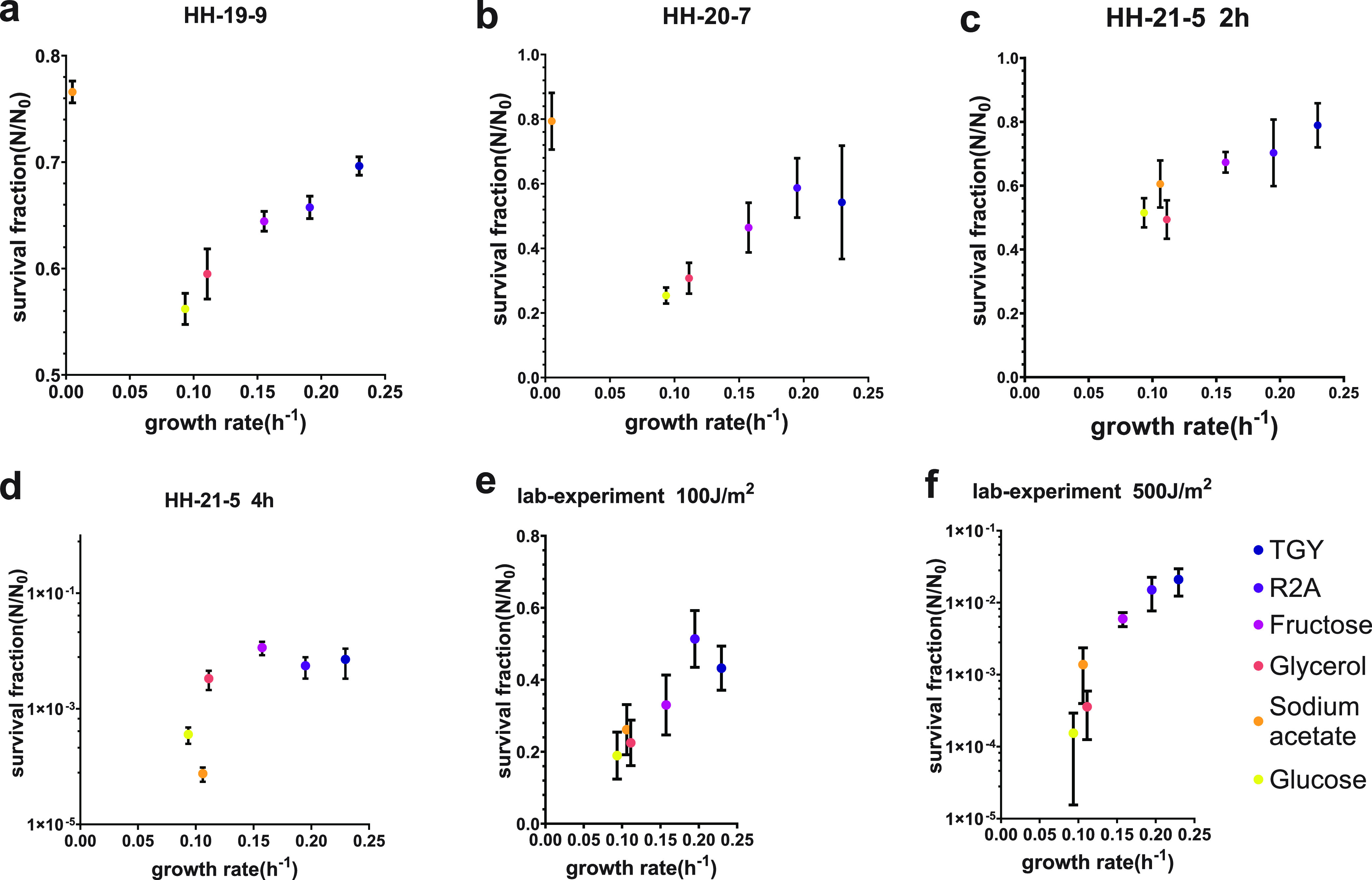
Results of experiments on different growth rates. (a to d) Survival fraction of D. radiodurans after exposure in near space in experiments HH-19-9, HH-20-5, and HH-21-5. (e and f) Survival fraction of D. radiodurans after exposure to doses of 100 J/m^2^ and 500 J/m^2^ in the laboratory. Graphics indicate a positive correlation between survival and growth rate. In experiments HH-19-9 and HH-20-7, it was unusual that the D. radiodurans cultured with sodium acetate as a carbon source had particularly low growth rates (~0 h^−1^) and high survival rates after exposure (77% and 80%, respectively). In the process of sample preparation, we found that because the pH value of the culture medium reached 8, and D. radiodurans could hardly grow in the medium with sodium acetate directly added. Thus, D. radiodurans was not in the exponential phase but in the stationary phase. Bacteria in a stationary state tend to be more resistant to various stressful conditions and radiation (27, 33, 35, 37, 86–88).

In experiment HH-19-9, the samples were exposed in near space for 2 h 22 min; D. radiodurans previously grown in different culture environments showed different sensitivities to near-space environments (Fig. 2a). The highest resistance, that about 7/10 of cells were alive after exposure, was exhibited by the D. radiodurans previously grown in casein tryptone-glucose-yeast extract (TGY) medium with a growth rate of 0.23 h^−1^, which was the fastest among all the media we used. Concurrently, the minimal medium with glucose as carbon source provided the lowest growth rate, 0.09 h^−1^, and the survival rate of bacteria previously cultured in this medium was the lowest, with an average of about 56%. The other three media (R2A and minimal media with fructose and glycerol) provided moderate growth rates (0.20 h^−1^, 0.16 h^−1^, and 0.11 h^−1^, respectively). The bacteria previously cultured in these media showed moderate persistence under near-space exposure (66%, 64%, and 59% survival, respectively). These results show that even for the same kind of bacteria, the resistance to exposure increased with the growth rate in preculture.

In experiment HH-20-7, the samples were exposed for 7 h 16 min in near space in order to determine the effects of longer exposure on the survival of bacteria (Fig. 2b). According to the data provided by the BIOSEP equipment provider, the diffuse UVC radiation dose received by samples during the direct exposure was 1.52 × 10^2^ J/m^2^, which was the only data available in the three flight missions because only the measurement of experiment HH-20-7 succeeded (57). However, we found survival rates similar to those in experiment HH-19-9 in this experiment. Many factors, such as the relative position of the balloon and the equipment, as well as the angle relative to the solar light, affect the actual exposure of the sample. Meanwhile, the flight altitude of experiment HH-9-9 was 32 km and that of experiment HH-20-7 was 23 km. These factors may have caused the total radiation intensities of the samples to be similar in the two experiments, resulting in similar survival rates. The difference between the two groups of experimental results was that the highest tolerance was provided by R2A medium in experiment HH-20-7 (about 59% survival on average), while experiment HH-9-9 was with TGY medium. In experiment HH-20-7, the average survival rate of bacteria previously grown on TGY medium was about 54%, close to the maximum value of 59% in this group, which was provided by bacteria cultured in R2A medium. Meanwhile, the most sensitivity was still exhibited by the bacteria cultured in minimal medium with glucose, with a cell survival rate of only 25%, which was consistent with the previous experiment. Moreover, the survival rates of bacteria previously cultured in minimal media containing fructose and glycerol in experiment HH-20-7 were 46% and 31%, respectively. Although the survival rate of the TGY group was 4% lower than that of the R2A group in experiment HH-20-7, it was still much higher than others. Thus, the results can still be roughly summarized as that resistance to near-space exposure is positively correlated with growth rates.

In experiment HH-21-5, we added another BIOSEP device to CAS-BAP that could be independently controlled; that is, we had two devices with different exposure times to determine the effects of different exposure durations under the same weather conditions in experiment HH- 21-5. Meanwhile, the samples of D. radiodurans were divided into two groups and exposed for 2 h and 4 h, respectively (Fig. 2c and d).

In the 2-h-exposed group, bacteria in TGY medium showed the highest tolerance: 79% of them subsisted. The two groups of bacteria with glycerol and glucose as single carbon sources had the lowest resistance, and their survival rates were 50% and 51%, respectively, with only a slightly difference between them. As expected, we obtained data for bacteria normally grown in medium with sodium acetate as the carbon source (Fig. 2d and Materials and Methods), with a growth rate of 0.11 h^−1^ and a survival rate of 61%. This is consistent with the relationships shown by bacteria grown with other carbon sources. In the 4-h-exposed group, the relationship between the resistance to exposure and the type of culture medium was the same as before, that is, the faster the growth rate, the higher the survival rate. Only 1.1% of the fructose group subsisted after 4 h of exposure, although this was the maximum survival in the 4-h-exposed group. Meanwhile, long-term exposure in near space appeared to be lethal to the bacteria grown in minimal media containing glucose or sodium acetate, and the survival rates were only 0.36‰ and 0.08‰, respectively, which were almost the detection limit of dilution plate method we adopted.

According to the coarsest theoretical estimate, the rate of bacterial survival of a certain condition in the 4-h-exposed group should be the square of the survival rate for corresponding one in the 2-h-exposed group. However, the actual result was much lower than this value. The reason was that the radiation intensity in near space did not vary uniformity with exposure time, and the UV dose of the 4-h-exposed group was unequal to twice that of the 2-h-exposed group. The exposure of the two groups started almost at the same time and lasted for different periods of time. The 2-h-exposed group started at 9:46 a.m. (Beijing time [BJT]) and ended at 11:46 a.m. Meanwhile, the 4-h-exposed group started at 9:49 a.m. and ended at 13:49, during which it experienced a noon of intense radiation. Therefore, the radiation dose of the 4-h group was much more than twice that of the 2-h group. It was understandable that the survival rate of the 4-h group was much lower than the theoretical estimates based on the exposure duration. A similar phenomenon was also observed in the experimental results for paraquat and Mn^2+^ in experiment HH-21-5, which could be explained by the same reason.

UVC is one of the main causes of cells death in near space, causing substantial damage to DNA, such as double-strand breaks (DSBs) (32, 60). The parameters of UVC during exposure were diﬃcult to determine precisely because the BIOSEP is swayed and rotated by the airflow, causing the sensor and the sample to sometimes not be in the same radiation environment due to their positions not coinciding. Furthermore, environmental conditions in near space were variable due to season, weather, and time of day, which makes the parameters diﬃcult to control for different missions. Therefore, we performed several experiments more precisely using the same protocols in the ground-based laboratory to verify the trends observed in near-space experiments using lab-generated UV light rather than near-space radiation (Fig. 2e and f). According to the data provided by the BIOSEP equipment provider, the UVC dose was only successfully measured in experiment HH-20-7, and the value was 1.52 × 10^2^ J/m^2^ (57). They estimate that the dose of the four exposure experiments was between 1 × 10^2^ J/m^2^ and 3 × 10^2^ J/m^2^. Considering that the dose range of laboratory experiments should be wider than for flight experiments and that it was diﬃcult to observe the death of D. radiodurans when the dose was less than 1 × 10^2^ J/m^2^ (48), we chose 1 × 10^2^ J/m^2^ and 5 × 10^2^ J/m^2^ UVC as our laboratory experimental radiation doses.

Under the 1 × 10^2^-J/m^2^ irradiation, the survival rate and the growth rate remained positively correlated, supporting the relationship we previously observed in near space exposure experiments. In the 5 × 10^2^-J/m^2^ irradiation experiment, the difference in the survival rates of bacteria cultured in different media became larger, as in the case of experiment HH-21-5. Combining near-space as well as ground-based laboratory experiments, we concluded that the survival rate of D. radiodurans after exposure, that is, the resistance to radiation, became higher with increasing growth rate, if different growth rates were caused by different nutrients.

**FIG 3:**
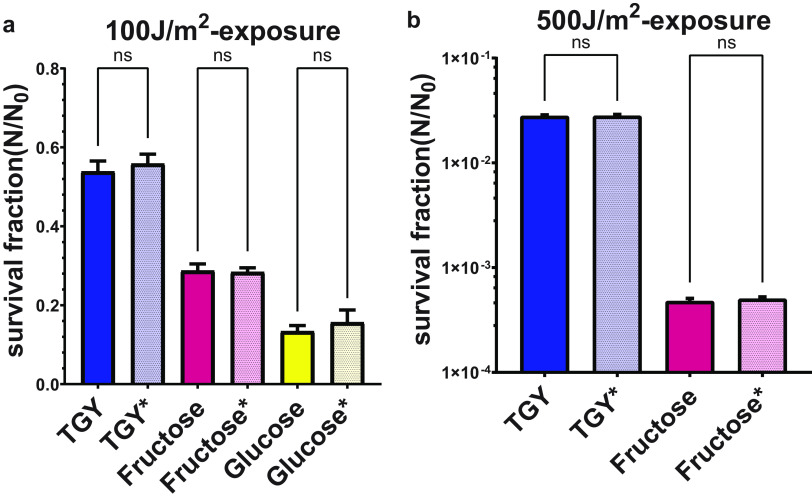
Results of comparative experiments with and without washing before lyophilization. We chose a rich medium and two minimal media to perform the experiments. Asterisks indicate that bacteria were washed before lyophilization. It could be observed that there was almost no difference between wash and nonwash groups after exposure to doses of 100 J/m^2^ and 500 J/m^2^. ns, not significant.

In order to clarify the effect of solids in the medium lyophilized with bacteria on the UV resistance of the samples, we performed comparative experiments using samples prepared by two different procedures, with and without washing prior to lyophilization. The results indicated that there was no significant difference in the UV resistance of D. radiodurans samples prepared by the two processes. This suggests that the difference in UV resistances of bacteria cultured in different media was due to the physiological discrepancies rather than the shielding of UV light by the different solid contents of the media (Fig. 3).

It seems that this phenomenon may be due to the fact that there is more DNA replication in bacteria at a high growth rate (36, 44, 61), which improves homologous recombination repair and thus enhances radiation resistance (62, 63). A large amount of UV radiation in near space, especially UVC, can cause UV-induced DNA damage, e.g., the photoproduct cyclobutan pyrimidine dimer (CPD), single-strand breaks (SSBs), and DSBs (32, 64, 65). The excision repair systems, consisting of base excision repair (BER), nucleotide excision repair (NER), and recombinational repair, are essential for bacterial survival (32, 65). Homologous recombination repair is one of the most important processes involved in DNA repair, ensuring that correct genetic information is transmitted from parents to offspring (32, 65). The success of repair could be affected by DNA content, that is, the number of gene replications (32, 64, 65). Many studies have shown that the abundance of DNA per cell increases exponentially with the growth rate, influenced by the quality of the available nutrients (36, 44, 61). This implies that for fast-growing bacteria, UV-induced damage would have more chance of being repaired by homologous recombination, suggesting that the high growth rate of exponential growth phase reduces the sensitivity of bacteria to UV radiation (63, 66).

### Growth in medium with paraquat decreases the sensitivity of D. radiodurans to near-space exposure.

The lethal damage induced by UVC includes not only DNA damage but also radicals and oxidants. Living in an environment of oxidative stress may have effects on cellular tolerance to radiation (32). We were curious about whether the oxidative environment that bacteria experience before entering near space helps them better cope with UV radiation, thereby improving their survival rate in near space. In order to investigate the effect of the previously experienced oxidative environment on bacterial sensitivity to near-space exposure, we added trace amounts of paraquat methosulfate (PQ; 10 μM added), a drug providing oxidative stress (67), to the several media we chose and prepared samples and conducted experiments following the same procedure as before. We compared the survival rates of bacteria that had lived in medium with and without added oxidants after near-space exposure.

**FIG 4:**
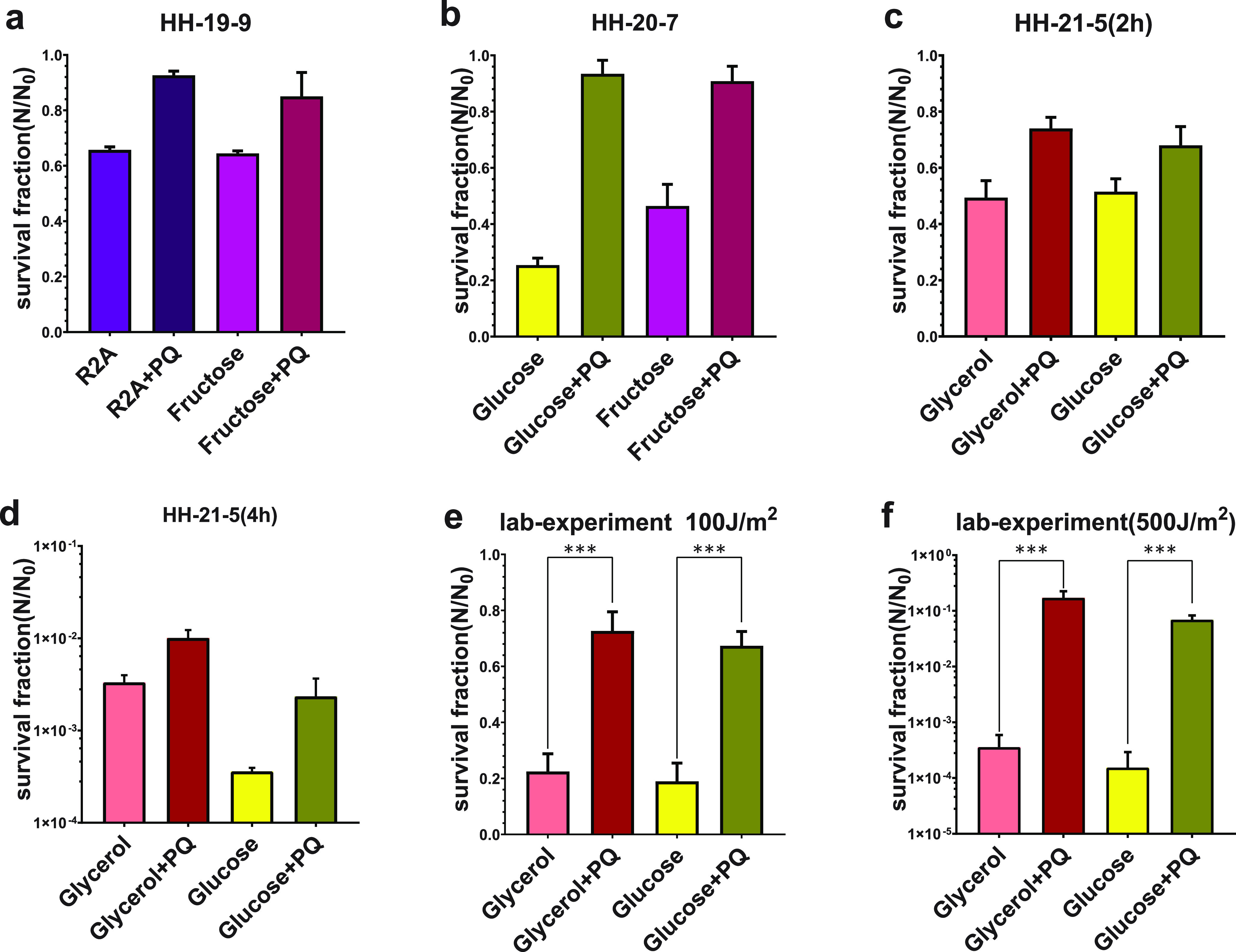
Results of experiments with paraquat. (a to d) Survival fraction of D. radiodurans after exposure in near space in experiments HH-19-9, HH-20-5, and HH-21-5. (e and f) Survival fraction of D. radiodurans after exposure to doses of 100 J/m^2^ and 500 J/m^2^ in the laboratory. Charts show that growth with paraquat increases the survival of D. radiodurans after exposure. ***, *P* < 0.001.

First, a rich medium and a minimal medium were selected to investigate whether the paraquat contributed to bacterial survival under exposure in experiment HH-19-9. The results showed that the addition of paraquat to the R2A medium and minimal medium with fructose increased the survival rate of D. radiodurans to 93% and 85%, respectively, which corresponded to increases of 0.4- and 0.3-fold (Fig. 4a).

Then in experiment HH-20-7, the fructose group and glucose group, the fastest- and slowest-growing bacteria in the minimal media we used, were used to investigate the effect of paraquat (Fig. 4b). Bacteria in PQ-added groups had survival rates of 91% and 93%, which indicated that they were 93% and 267% more tolerant to near-space exposure, respectively, than the non-PQ-added groups.

In experiment HH-21-5, two minimal media yielding similar growth rates using either glucose or glycerol as the carbon source were chosen with the purpose of verifying the effect under different exposure durations. Adding paraquat to medium with glucose or glycerol displayed similar resistance improvement in short exposure of experiment HH-21-5, 0.3- and 0.5-fold increases, reaching 68% and 74%, similar to the case with experiment HH-19-9 (Fig. 4c). Comparing with previous results, we found that in the 4-h exposure of experiment HH-21-5, the survival rate of the glucose group and the glycerol group reached 10.00‰ and 2.34‰, increases of 6.5-fold and 3-fold, respectively (Fig. 4d). We also performed more precise and controllable experiments in the ground laboratory using UV lamps instead of near-space exposure, and the results showed a similar trend: that paraquat could increase the resistance to radiation (Fig. 4e and f). Therefore, we propose that experience of growth under oxidative stress is beneficial for bacterial populations to improve resistance to radiation or near-space exposure.

It was reasonable to deduce that bacteria express antioxidant proteins and factors during growth in medium containing oxidants, and increase their resistance to UVC, because the oxidative stress is another form of lethal damage caused by UVC. Reactive oxygen species (ROS) or oxidative stress induced by radiation leads to oxidation of nucleotides, proteins, and the lipid membrane, causing the disruption of cellular redox homeostasis. These forms of damage last for a substantial time after exposure and may lead to decreases of genomic stability, alterations of gene expression, and elevated mutagenesis rates, resulting in changes in cell survival and proliferation (50, 51, 68–70). There is documented evidence that cell death strongly correlates with proteome carbonylation (PC) caused by ROS (50, 51, 71, 72). The presence of a biological response to cellular damage or protection against molecular damage, such as that by ROS, ensures the survival of bacteria exposed to radiation (71, 75). According to published literature, numerous antioxidant proteins, e.g., superoxide dismutase (SOD), OxyR, PerR, DdrI, and PprI, as well as various antioxidant metabolites against ROS and PC, help to enhance protection of the proteome of D. radiodurans (50). Moreover, some studies showed that many small RNAs also play essential roles in the transport of antioxidant proteins and regulation of manganese and DNA repair systems in D. radiodurans (73, 74). Some proteins reduced the effect of oxidized RNA, which also helped protect cells from oxidative stress (75). Growing in stress environments may upgrade these factors, which help survival in exposure.

Since both UVC and paraquat provided oxidative stress (65, 67), we speculated that bacteria grown in an oxidative environment had expressed antioxidant proteins and antioxidative metabolites to increase antioxidant capacity, which helped to cope with oxidative damage caused by UVC (50).

### Mn^2+^ increases the resistance of D. radiodurans to near-space exposure.

Manganese is thought to be an important element for organisms, especially essential for metabolism and the antioxidative system (76, 77). Manganese is mainly present in cells in the form of divalent ions, and it is a key component of various enzymes, including hydrolases, transferases, and oxidoreductases (50). Many studies have shown that the radioresistance of several bacteria correlates with concentration ratios of intracellular Mn^2+^ and Fe^2+^ (51, 78, 79). If the bacteria had previously grown in an environment rich in Mn^2+^, would the survival rate be different when they entered near space? Hence, it is necessary to study the effect of manganese and further discuss the memory effect of growth conditions on near-space exposure. Like when studying the effects of paraquat, we chose the same medium and followed the same procedure to study the role of manganese in resisting near-space exposure.

We explored the effect of Mn^2+^ on bacterial survival in experiment HH-19-9 and further validated it in the next two missions. Manganous acetate was added to the selected medium until the concentration of Mn^2+^ was 50 μM to elevate the value of Mn^2+^/Fe^2+^.

**FIG 5:**
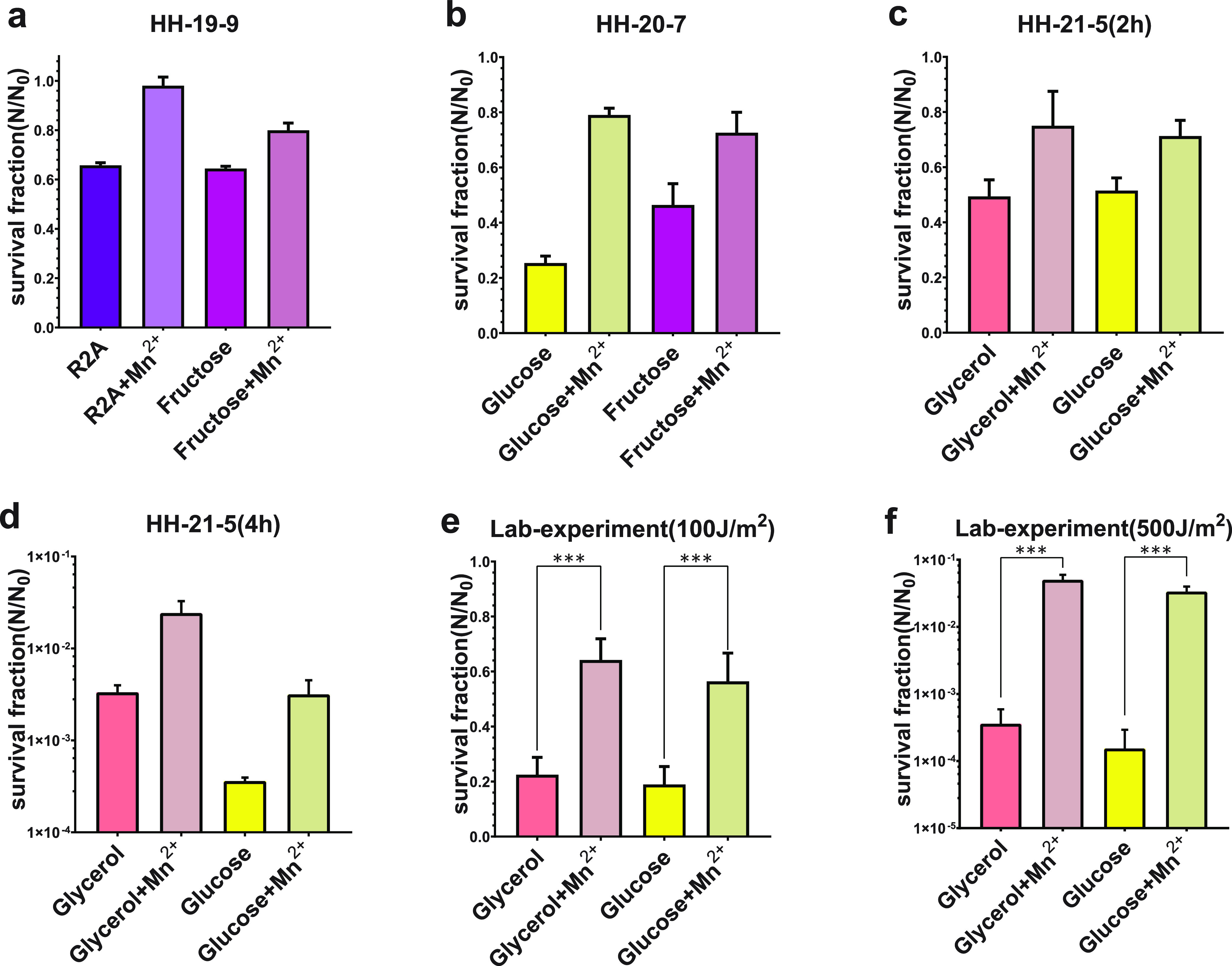
Results of experiments with Mn^2+^. (a to d) Survival fraction of D. radiodurans after exposure in near space in experiments HH-19-9, HH-20-5, and HH-21-5. (e and f) Survival fraction of D. radiodurans after exposure to doses of 100 J/m^2^ and 500 J/m^2^ in the laboratory. Charts show that Mn^2+^ increases the survival of D. radiodurans after exposure. ***, *P* < 0.001.

It turned out, as expected, that the survival rate increased with the increase of Mn^2+^/Fe^2+^ in all cases. In the results of experiment HH-19-9 (Fig. 5a), bacterial survival in the R2A group and fructose group increased to 98% and 80% under the influence of high concentrations of manganese, which corresponded to increases of 0.5- and 0.24-fold, respectively, compared with the normal groups. Experiment HH-20-7 showed 0.6- and 3.1-fold increases in the fructose group and glucose group, and their survival rates reached 73% and 79% (Fig. 5b). The most amazing results were observed with 4-h exposure in experiment HH-21-5: the survival rates of the glucose group and glycerol group increased to 3.16‰ and 24.32‰, with an 11-fold increase in bacterial survival in the glucose group and a 7.4-fold increase in the glycerol group (Fig. 5c and d). Using artificial UV illuminant, we have carried out accurate and controllable experiments in the laboratory and found that Mn^2+^ can improve the survival rate of bacteria under UV irradiation, as shown in near space (Fig. 5e and f). The results indicated that the high level of manganese did enhance the resistance to radiation and near-space exposure, which was consistent with previous reports (51, 79).

The mechanism of Mn^2+^ has been demonstrated by X-ray fluorescence microspectroscopy; manganese is usually colocalized with the DNA-containing nucleoid and acts as an antioxidant near the genome, where DNA repair and replication proteins are most needed (68, 72, 80). A delicate manganese regulatory system consisting of several types of manganese transporters has been found in D. radiodurans; this system supports the cellular uptake of suﬃcient manganese and maintains high levels of Mn^2+^ in the cell (81, 82). Moreover, the PprM (DR0907), OxyR1 (DR0615), DtxR (DR2539), and Mur (DR0865) proteins are also involved in the regulation of manganese homeostasis. High concentrations of manganese in the environment offer more synthesis of antioxidant enzymes and factors, thus helping to maintain the normal operation of repair and antioxidant responses (50). Therefore, the proteins involved in the antioxidative system and repair system were highly expressed in bacteria cultured with a high concentration of Mn^2+^. This improved antioxidant capacity and DNA repair ability and thus increased the resistance to UVC (50).

## DISCUSSION

We performed exposure experiments in near space and the laboratory with lyophilized D. radiodurans in exponential phase. We observed that the resistance of D. radiodurans to near space or UVC radiation increased as the growth rate increased. It was reasonable to deduce that fast-growing bacteria had more DNA replication, which led to an increase in radiation resistance (36, 44, 61). Then we noticed that the stressful environment also had memory effects on the radiation response. Paraquat and Mn^2+^ enhanced radioresistance of D. radiodurans, which may be because the antioxidative system was activated before radiation (51, 78, 79).

Many researchers have carried out experimental studies on the relationship between growth rate and stress resistance with different methods. Some of them found a phenomenon similar to ours, in which fast-growing bacteria showed higher resistance (33–35, 83). However, there are other researchers whose results are different from ours (40, 84). Berney et al. performed an exposure experiment with UVA, and the relationship between radiation resistance and growth rate was just the opposite of ours (84). We suspected that the one of the reasons for the inconsistency was the different types of DNA damage caused by UVA and UVC. UVA mainly causes the photoproduct cyclobutan pyrimidine dimer (CPD), which can be removed only by photoreactivation. Fast-growing cells contain more DNA, which means that more UVA-induced CPDs need to be repaired, because the probability of damage to each base per unit dose of UVA is the same (30, 32, 65). Therefore, it would be a huge burden to remove the CPD for these fast-growing cells. They will die when CPD cannot be repaired in time due to lack of photolyase (85). UVC and ionizing radiation cause double-strand breaks (DSBs), and more DNA is helpful to repair the DSBs through homologous recombination. Thus, rapid growth is beneficial to survival under UVC and ionizing radiation.

**FIG 6:**
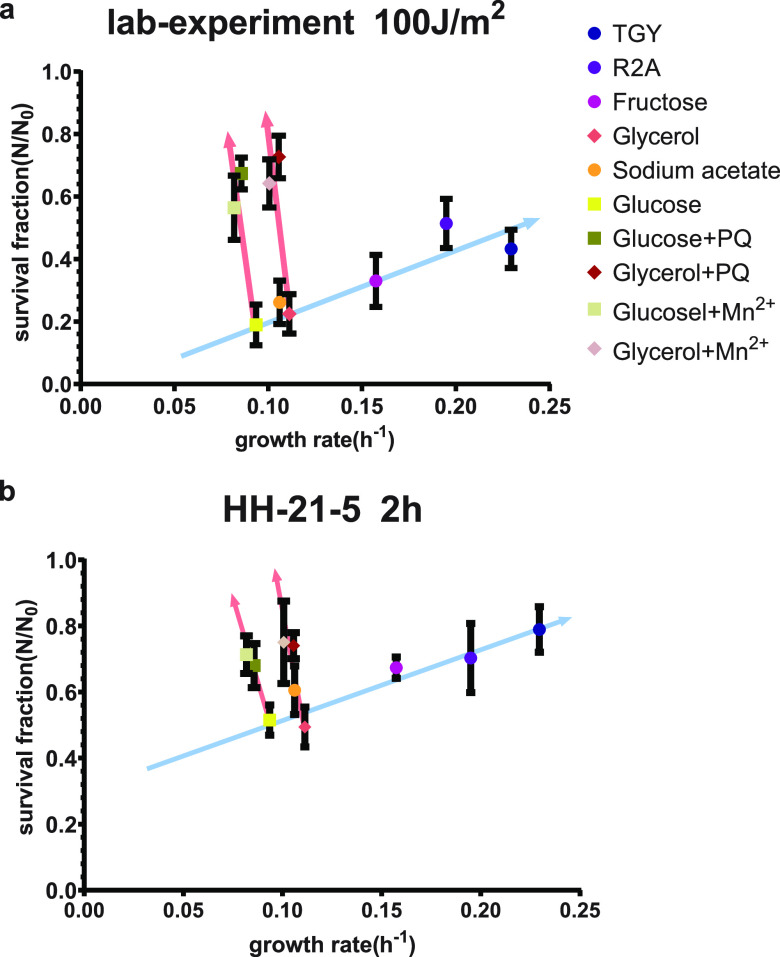
Comparison of all groups in one experiment. (a and b) Experiments with 2-h exposure in HH-21-5 and a dose of 100 J/m^2^ in the laboratory shown as examples. The blue arrow indicates the positively correlation between survival rates and growth rates if the differences in growth rate are caused by differences in nutrition. The red arrows show the trend that radiation resistance would be enhanced as growth rate decreases due to stress. Trend lines were fitted using the least square linear fitting. (For panel a, blue arrow, *y* = 2.1064*x* + 0.3383 and *R*^2^ = 0.827; red arrows, left *y* = −32.982*x* + 3.3596 and *R*^2^ = 0.5907 and right *y* = −32.26*x* + 3.9192 and *R*^2^ = 0.5784. For panel b, blue arrow, *y* = 1.9567*x* + 0.3383 and *R*^2^ = 0.8765; red arrows, left *y* = −45.223*x* + 4.4815 and *R*^2^ = 0.8335 and right *y* = −40.916*x* + 4.8688 and *R*^2^ = 0.7436.)

In addition, we controlled the growth rate by using different carbon sources rather than by changing the concentration of carbon source as Berney et al. did (84). We used saturated concentrations of nutrients without stress to allow D. radiodurans to grow at the maximum growth rate of this medium, which ensured that the bacteria were in the optimal physiological state in this environment and stress response proteins were largely unexpressed. However, controlling the bacterial growth rate by adjusting the dilution rate of the thermostat will result in additional stress on cells, such as starvation and metabolic waste, when low growth rates are needed (36). With the second method it is diﬃcult to separate the effect of some external stress on bacteria at different growth rates, and it is also diﬃcult to determine the effects of different DNA contents on bacterial radiation resistance. This inspired us to observe how a decrease of growth rate caused by stress affects bacterial resistance to UV radiation. We plotted the survival rates of normal groups, paraquat-added groups, and Mn^2+^-added groups based on the same medium in one graph. As is shown in Fig. 6, the slower growth caused by stress improved survival from exposure, which is consistent with the findings of Berney et al. (84).

Bucheli-Witschel et al. proposed that the relationship between UVC resistance and antecedent specific growth rate may be the result of several mechanisms that affect the sensitivity of bacteria to UVC depending on the growth rate (35). Some of these factors increase the tolerance of UVC, while others offset the effect (85). There was evidence that very-slow-growing cells were more resistant to stress than normal-growing cells due to increased levels of *rpoS* expression and SOS responses, which helped to adapt to stress and repair the radiation-induced DSBs (37, 40–42, 79).

Our results suggest that bacteria may have an advantage in dealing with near-space exposure if they have previously grown in environments that support faster growth or have experienced associated stress environments, such as oxidative stress. As a result, they can migrate through the stratosphere with higher survival rates, thereby expanding their distribution on Earth (8). Organisms have been attempting to improve growth rates in order to gain a growth advantage, but expanding habitat to avoid local competition is equally important for subsistence. Our research shows that evolution aimed at these two directions is complementary rather than conflicting. Furthermore, we could utilize the correlations of various stress environments and memory effects to conduct laboratory evolution experiments, for example, in the use of oxidative environments to help obtain highly UV-resistant strains. The discovery of various memory effects may also be of great significance to understand the survival strategies of microorganisms in extreme environments.

## MATERIALS AND METHODS

### Strain information.

The wild-type strain we used in this study was Deinococcus radiodurans CGMCC 1.633, purchased from China General Microbiological Culture Collection Center (CGMCC).

### Growth media and cultivation conditions.

The growth media used in this study can be grouped into two categories—minimal media and nutrient-rich media—and all of them can provide different growth rates for D. radiodurans. All the minimal media were based on the basal salt medium for D. radiodurans used by Venkateswaran et al. (58), with slight modifications.

Casamino Acids (0.2% [wt/vol]) were provided as a nitrogen source. One of the following substrates was used as the carbon source for each growth condition: 0.2% (mass/vol [m/v]) fructose, 0.2% (m/v) glycerol, 0.2% (m/v) glucose, and 0.2% (m/v) sodium acetate. For rich media, we used TGY medium and R2A medium to obtain a higher growth rate than in the minimal media. Moreover, we added 10 μM paraquat methosulfate in each of the minimal media for the oxidative stress group, and 50 μM manganous acetate in total was provided for the Mn^2+^-added group. The details of all media and growth conditions are listed in Table 1.

All groups of D. radiodurans organisms were grown in different media within a 28°C shaker at 230 rpm.

In experiments HH-19-9 and HH-20-7, 0.2% (m/v) sodium acetate was added to the bacterial preculture medium with an optical density at 600 nm (OD_600_) of 0.5 and then cultivated for more than 1 h, in order to understand the effect of sodium acetate as a carbon source on sensitivity of D. radiodurans to near-space exposure. The growth of D. radiodurans stopped immediately after the addition of sodium acetate. In experiment HH-21-5 and laboratory experiments, we changed the protocol and used sodium dihydrogen phosphate solution, an acidic weak liquid, to adjust the pH value of the medium to 7.

### Preparation of sample.

Precultures were prepared for each individual batch experiment from the same cryovial stored at −80°C by streaking onto R2A agar plates. For batch cultivation, a single colony from an R2A plate which underwent 96 to 120 h of incubation at 28°C was transferred into 6 mL of preculture medium (R2A or glucose minimal medium) in a sterile 10-mL plastic tube and incubated overnight at 28°C with agitation at 230 rpm. Then we inoculated the preculture into fresh, prewarmed batch growth medium in an Erlenmeyer flask to give an initial OD_600_ of ca. 0.01 in the case of the media mentioned above. Cells were grown in a 28°C shaker at 230 rpm, and OD_600_ was measured to obtain the growth curves and growth rates of exponential growth under all cultivation conditions. The cell culture at exponential phase was then taken out as a sample.

Sterilized quartz 96-well plates, which enabled >86% transmission of UV at 200 to 280 nm, were used as sample holders. A portion of bacterial culture in each growth condition whose OD_600_ was between 0.4 and 0.5 was dropped into 96-well plates (Fig. 1b), and to each well was added 50 μL or 200 μL of bacterial liquid. Four parallel samples were prepared for each growth condition, in order to avoid errors and eliminate deviation, and all kinds of growth condition could be found in one plate. We prepared several 96-well plates that were the same as for exposure group and nonexposure group in the experiments, and the samples in corresponding positions of different plates were from same culture (Fig. 1c).

Following the completion of the bacterial liquid transfer, samples were lyophilized at −80°C under vacuum immediately for 36 h and turned into a thin layer attached to the bottom of the wells (Fig. 1d). The measurement of OD_600_ continued several times after sampling to make sure the cells we harvested were taken during exponential growth. In the near-space experiments, the lyophilized samples were stored at −20°C after preparation and were transported to the experimental site. In the laboratory experiments, exposure was performed immediately when the freeze-dried sample was prepared.

### Experiment process.

Samples were divided into two groups: an exposed group and a nonexposed group. In the near-space exposed experiment, the lyophilized samples were both fixed on the Biological Samples Exposure Payload (BIOSEP) designed by CAS and flown with CAS-BAP to conduct the exposure experiment (Fig. 1d). The plate was mounted bottom up, in order to make the sample better able to receive radiation, because the lyophilized samples were attached to the bottom.

During the flight, the samples remained shielded inside the BIOSEP before and after the exposure. Upon reaching a predetermined altitude, the CAS-BAP turned to level flight, and the box cover of BIOSEP was opened by computer remotely for a predetermined period of time for exposure. The nonexposed group was shielded from radiation by a baffle even when BIOSEP was in open mode. Then CAS-BAP landed at the location we planned. After the near-space flight, the sample plates were wiped with alcohol and saved at −20°C until the activation of experiment. We conducted three near-space exposure experiments at Dachaidan, Qinghai, China (37°44′N, 95°21′E) in total, named HH-19-9, HH-20-7, and HH-21-5. The data of the flight and the strength of UV radiation were measured by the sensor of BIOSEP and provided by BIOSEP’s design agency.

The laboratory experiments were performed in the radiation device, which contained UVC lamps (provided by Shanghai Shiping Experimental Equipment Co., Ltd.; rated power is 8 W and irradiance of UVC is 71.6 mW/m^2^ at a distance of 30 cm from the lamp), to replace the exposure of near-space UV radiation. The samples were exposed for 23 min and 116 min, respectively, also with the nonexposed group to determine the rates of survival of irradiation.

Because the temperature curves of the four flight experiments were greatly different and fluctuated between –40°C and 50°C, we chose 20°C, which was close to the average value of temperature curves in flight experiments, as the experiment temperature when samples were exposed to UVC.

To ensure reproducibility, the experiments in the laboratory were repeated three times and used the same dose of UVC radiation. Descriptive analysis and tests of statistically significant differences were adopted in the final result analysis. The average results of three experiments are exhibited.

### Statistics of survival rates.

A total of 200 μL of basal salt medium without carbon source or nitrogen source was used to resuspend the freeze-dried samples. The suspensions were then decimally serially diluted to a final dilution factor of 10^−7^. A required volume of the suspensions was spread over the R2A agar plates and incubated for 120 h at 28°C to determine the survival number of each sample, because the number of survivors in each sample was estimated by CFU enumeration. The number of surviving cells of each sample in unit volume can be calculated as *N_i_* = *n* × *d*/*V*, where *n* is the average count of colonies on plate, *d* is the dilution factor, and *V* is the volume of the bacterial suspension spread. And then the survival rates of each sample after exposure of different irradiation conditions can be derived from *S* = *N_i_*/*N*_0_, where *N_i_* is survivor number of each growth condition after irradiation and *N*_0_ is the survivor number of the nonirradiated sample in the corresponding growth condition.
